# Foot- and Mouth-disease

**Published:** 1897-11

**Authors:** 


### ABSTRACTS FROM FOREIGN JOURNALS.

### GERMAN.

UNDER THE DIRECTION OF J. PRESTON HOSKINS,
PRINCETON, N. J.

FOOT- AND MOUTH-DISEASE.—Just as one year ago, the foot- and mouth-disease has broken out again in its severest form, and has already worked considerable damage. The course of the disease is again such that the local trouble on the mucous membrane of the mouth and hoofs is usually in process of healing when the cattle are seized with sudden and severe disturbances in their general condition, and after a very short time death ensues.

In regard to the cases which ended fatally, the reports have been, almost without exception, that the animals were very severely afflicted with foot- and mouth-disease. This could be seen in the almost complete loss of appetite, the failure to secrete milk, the very significant state of exhaustion and lack of interest, as well as in the high eruptive fever which sets in very frequently with a violent chill. Yet such severe cases occurred often enough without death or other especial complications following in the course of the disease. The exanthema (or eruption) in the cases which ended fatally showed absolutely no difference from the normal phenomena, and neither from its appearance nor its course in itself, nor the number, size, and character of the efflorescences, could any data be obtained which would enable one to determine the fatal cases as such.

A striking increase in the pathological changes could certainly not be proven. The course of the disease was, as a rule, that the bosses of matter, after the bursting of the blebby efflorescences, began to heal over in the fatal cases as well as in those which recovered. The edges were smooth, and a layer of new-formed epithelium or fresh epidermis could plainly be seen. With the continuance of the healing process at the localizations, the general condition of the animal improved. Appetite and rumination returned, and the secretion of milk began again gradually. In individual cases the fact was especially emphasized that the desire for food was extraordinarily abnormal; a fact which received full confirmations from the conditions present on opening the first stomach or paunch. In this far-advanced stage of the disease the animals were either very suddenly overtaken by death, or, after severe and sudden changes for the worse in the general condition, succumbed in the course of a few days. The sudden change for the worse showed itself again in the loss of appetite, in the state of exhaustion, and by a striking difficulty in breathing (dyspnœa). Yet this relapse was not accompanied by feverish symptoms, as Imminger has already observed. In all cases death followed pretty directly, the animals simply collapsing or tumbling over and dying quickly.

All these phenomena are not new, and simply agree with the observations which have been made here in Lower Austria in former years. I will add, however, that experience here in Lower Austria, as well as in Bavaria, in the severe cases goes to show that the care which the animals are wont to receive seems to have no influence on the course of the disease described. The severe and fatal course of the disease occurs as often in stalls which are kept with the greatest cleanliness and enjoy the best hygienic conditions as in those which are poorly situated, damp, with poor ventilation, and no attention.

I will now touch upon the data which were obtained by a post-mortem examination. The results show some peculiarities. Beside the loss of material in the healing process of the mucous membrane of the mouth and on the hoofs, there was almost in all cases the symptoms of an œdema of the lungs, and besides changes in the first stomach constantly present, while the changes in the other divisions of the stomach as well as in the intestine were very slight, and in individual cases failed entirely. The inflammatory œdema of the mucous membrane of the rennet (fourth stomach), as has been described by Kitt, could not be discovered in any case. The symptoms of a gastro-enteritis in the rennet and intestine, as had been observed in the previous year, were present in only one case. In this case the contents of the rennet and of the smaller intestine were of a blood-red color. Splenic tumors were absent, and no symptoms of disease could be discovered in the heart. As regards the paunch, there was found in it thickly coagulated efflorescences of a circular form, which were all in the process of healing up. The edges were flat, surrounded with new-formed epithelium; but in the middle they were of a brownish-red color; the epithelium on the rest of the paunch was more easily peeled off than under normal conditions. The mucous membrane beneath was deeply reddened. In places large circular spots were noticeable, on which there was no epithelium. The mucous membrane here appeared swollen and had a brownish-red color.

The essential point, however, seems to me to be the sudden sinking and death while the efflorescences are in process of healing up. In no case was it possible to find a fresh or even an eruption (pustule) in an advanced state. Everywhere they were open and in process of healing up.

In these cases the question is, How are the general disturbances or death to be accounted for? Clinically it makes the impression as if poisoning had taken place. The many theories which have been advanced heretofore for foot- and mouth-disease do not seem to me applicable here. The assumption of a destructive virus, however, is only a circumlocution, and proves nothing; just as little satisfactory is the theory that death is brought about by the production of poisonous matter on the part of the bodily infectious material which has found its way into the succum and sunguinem. The explanation of Willich seems more probable, namely, that the microorganisms which are found in the intestines under normal conditions, and which are not pathological when the mucous membrane is healthy, develop a pathological effect from the mucous membrane of the intestines when it is inflamed by the foot- and mouth-disease.

I think that the fatal outcome in the cases where changes were present in the paunch alone might be explained as follows: Normally a certain fermentation of decay runs off into the paunch. It is then not impossible that poisons may be generated. When the foot- and mouth-disease is localized in the paunch, the thick protecting layer of epithelium may be broken through by the eruption of so many pustules, and the poisonous stuff would find its way between the fibres of the walls, and by way of the lymphatic ducts find its way into the circulation and be the occasion of the very grave consequences which are so prominent in the acute form of the disease. However, by means of experiment, we must first determine whether poisons of such a nature are really to be found in the normal contents of the paunch, or are in that merely of fasting animals. Besides, another question arises, Why this disease at one time localizes itself in the mucous membrane of the mouth and the skin of the hoofs, and at other times appears in the paunch also.

In conclusion, Prof. Schindelka makes a plea for governmental indemnification to cattle-owners whose source of support is so often endangered by these pests. He is also in favor of making the insurance of cattle compulsory.—PROF. H. SCHINDELKA in *Thierarztliches Centralblatt*, November 1, 1895.

REGULATIONS FOR THE PREVENTION AND SUPPRESSION OF FOOT- AND MOUTH-DISEASE.—In consequence of the extraordinary spread of the foot- and mouth-disease, the question of the prevention of the epizootic has become intense, and the need of greater preventive efforts is more and more apparent. The writer has attempted to hinder the spread of the epizootic by means of the disinfection which was recommended by John in 1894. Only in my case the disinfection was applied to the infected herds also, first to destroy the virus and to make the transmission of it impossible, and then to bring about a rapid and complete healing up. The result was a very satisfactory one.

A 1 per cent. solution of crude solutol was applied. This preparation was especially recommended on account of its cheapness and because no other preparation of cresol in such a dilution produces an equal effect. A piece of cloth was soaked in the solution, and the mouths of the infected animals, as well as those which were suspected, were thoroughly swabbed out; then the whole body was wiped off with a damp cloth and the udder and hoofs thoroughly washed off. Then, with a sprinkling-can, all objects which could have been tainted with the flying spittle, or through contact with the tail, were wet with the solution. This application was repeated the following day, but in the meantime the udders and legs of the animals were frequently washed with the solutol solution. As a matter of course, the clothing and especially the shoes of the attendants were subjected to a thorough cleansing.

Although at first our only purpose was to hinder the transmission of the virus, yet it was very soon apparent that the severity of the infection had been diminished. The production of milk in stables which averaged 300 litres per day sank for only two days to 230 to 250 litres, and the appetite of the animals, with careful feeding, did not fall off. The long suffering from hoof-trouble, which often appears as a consequence of the disease, was entirely avoided. The epidemic remained confined to separate localities where the owners could be induced to adopt these measures. Often enough there was opportunity to notice the differences in the appearance of the disease between the disinfected stables and those which had been neglected. They showed clearly the value of our efforts.—*Idem.*

